# Optimization of Processing Parameters in ECM of Die Tool Steel Using Nanofluid by Multiobjective Genetic Algorithm

**DOI:** 10.1155/2015/895696

**Published:** 2015-06-18

**Authors:** V. Sathiyamoorthy, T. Sekar, N. Elango

**Affiliations:** ^1^Department of Mechanical Engineering, Dr. Navalar Nedunchezhiyan College of Engineering, Tholudur 606 303, India; ^2^Department of Mechanical Engineering, Thanthai Periyar Government Institute of Technology, Vellore 2, India; ^3^Department of Mechanical Engineering, UCSI University, North Wing, 56000 Kuala Lumpur, Malaysia

## Abstract

Formation of spikes prevents achievement of the better material removal rate (MRR) and surface finish while using plain NaNO_3_ aqueous electrolyte in electrochemical machining (ECM) of die tool steel. Hence this research work attempts to minimize the formation of spikes in the selected workpiece of high carbon high chromium die tool steel using copper nanoparticles suspended in NaNO_3_ aqueous electrolyte, that is, nanofluid. The selected influencing parameters are applied voltage and electrolyte discharge rate with three levels and tool feed rate with four levels. Thirty-six experiments were designed using Design Expert 7.0 software and optimization was done using multiobjective genetic algorithm (MOGA). This tool identified the best possible combination for achieving the better MRR and surface roughness. The results reveal that voltage of 18 V, tool feed rate of 0.54 mm/min, and nanofluid discharge rate of 12 lit/min would be the optimum values in ECM of HCHCr die tool steel. For checking the optimality obtained from the MOGA in MATLAB software, the maximum MRR of 375.78277 mm^3^/min and respective surface roughness Ra of 2.339779 *μ*m were predicted at applied voltage of 17.688986 V, tool feed rate of 0.5399705 mm/min, and nanofluid discharge rate of 11.998816 lit/min. Confirmatory tests showed that the actual performance at the optimum conditions was 361.214 mm^3^/min and 2.41 *μ*m; the deviation from the predicted performance is less than 4% which proves the composite desirability of the developed models.

## 1. Introduction

Advanced high hardness materials have a high importance especially for the applications such as automotive, metal forming, die making, and aerospace industries. ECM is more suitable process to have excellent and precise machining of these hard materials. It is a technical alternate in the field of manufacturing process to machine steels and superalloys due to avoidance of thermal stresses on their microstructures and absence of tool wear during the machining process [1, 2]. It is more appropriate one to machine a nonmachinable hard materials such as HCHCr die tool steel, AISI 202 Austenitic stainless steel, and superalloys [3, 4]. The parameters of the ECM influencing the objectives of MRR and surface roughness are applied voltage, tool feed rate, and electrolyte discharge rate [5–7]. The electrolyte flows through the interelectrode gap (IEG) and the machining reaction is very appreciable when the value of IEG is small [8, 9]. During the electrochemical machining, the formation of spikes, due to presence of passive layer formation, inconsistency of current density, and formation of gas at the IEG, prevent achievement of the better MRR and surface roughness. Hence this attempts to minimize the formation of spikes in the machined component by using copper nanoparticles suspended in NaNO_3_ aqueous electrolyte solution. In order to find out an optimal condition, multiobjective genetic algorithm (MOGA) has been applied in this research work.

## 2. Experimental Setup

**Figure 1:**
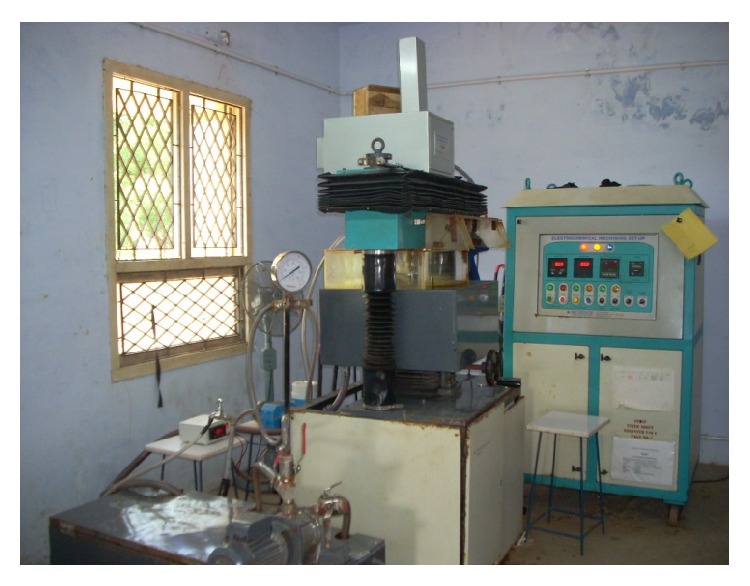
ECM setup.

The experiments were conducted using ECM setup as shown in Figure 1. The selected workpiece material HCHCr die steel with hardness of 67 in HRc scale was one of the poor machinability materials [10]. The complete chemical composition of HCHCr die steel is presented in Table 1. The plain aqueous solution of 15% NaNO_3_ and 40 g of Cu nanoparticles suspended in plain aqueous solution of 15% NaNO_3_ were selected as electrolytes in these experiments [11]. The electrolyte solution was completely analyzed using deluxe water and soil analysis kit, Model-191E. A digital flow meter with two-digit accuracy was employed to adjust the flow rate of electrolyte to the IEG. Copper was chosen for fabrication of tool due to high electrical conductivity. In the present work, the IEG is set to be 0.5 mm initially throughout the experimentation [12]. Material removal (MR) is the difference in the weight of the workpiece before and after machining. The accuracy of measurement is ensured using Sartorius electronic weighing machine with three-digit accuracy. Mitutoyo surface tester with a range of 0–150 *µ*m is used to measure surface roughness (Ra) and the average of values observed in three different surfaces on the workpiece is computed in each experiment. The process parameters used in the complete experiment are presented in Table 2.

## 3. Mathematical Modeling of Machining Parameters

Design Expert 7.0 software is used to determine the relationship among the selected influencing parameters. Three levels have been selected for influencing parameters of the applied voltage, electrolyte discharge rate, and four levels selected for tool feed rate. It is possible to assess the main and interaction effects of different machining parameters in L_36_ array with most reasonable accuracy. A first-order experiment was performed to determine the magnitudes of the relative changes to the process parameters that would result in optimum MRR and surface roughness. It is obtained from the first-order experiments; copper nanoparticles suspended in aqueous NaNO_3_ electrolyte significantly improve the MRR and surface roughness compared to plain aqueous NaNO_3_ electrolyte. Subsequently, a second-order central composite design was selected to identify the optimum conditions which turn into the higher MRR and finest surface roughness. The general form of second-order polynomial mathematical model applied to investigate the parametric effects of ECM is

(1) $$\begin{array}{c} Yu=b_{0}+\overset{n}{\underset{i=1}{\sum }}b_{i}x_{iu}+\overset{n}{\underset{i=1}{\sum }}b_{ii}{x_{iu2}}^{2}+\overset{n}{\underset{i<j}{\sum }}b_{ij}x_{iu}x_{ju}, \end{array}$$

where *Yu* is the response and terms *b*_0_, *b*_*i*_, and so forth are the second-order regression coefficients. Various sets of parametric combinations results are obtained by conducting a series of experiments. The respective mathematical models representing MRR in view of plain aqueous NaNO_3_ and Cu nanoparticles suspended in plain aqueous NaNO_3_ electrolyte are computed as

(2) YuMRR=−17.794−22.942X1−340.754X2 +54.275X3+6.834X1X2−2.018X1X3 +4.178X2X3+1.642X12 +280.163X2²−0.682X3²,

(3) Yu1MRR=−603.747+117.441X1−170.503X2 −48.750X3+12.903X1X2−0.215X1X3 +11.491X2X3−3.442X12 +98.005X22+3.305X32,

where *Yu*(MRR), *Yu*_1_(MRR), *X*_1_, *X*_2_, and *X*_3_ represent MRR of plain aqueous NaNO_3_, Cu nanoparticles suspended in plain aqueous NaNO_3_, applied voltage, tool feed rate, and electrolyte discharge rate, respectively. The developed mathematical model will enable improvement of the performance of ECM while machining HCHCr die steel. The degree of fitness of the developed mathematical model is confirmed through ANOVA test. The coefficient of determination *R* ^2^ for MRR in terms of aqueous NaNO_3_ and Cu nanoparticles suspended in aqueous NaNO_3_ solutions were 90.97% and 93.45%, respectively, which confirms the accuracy of fitness of the mathematical model.

The respective mathematical models representing surface roughness in view of plain aqueous NaNO_3_ and Cu nanoparticles suspended in plain aqueous NaNO_3_ electrolytes are computed as

(4) YuSR=−24.807+1.252X1+6.630X2+3.024X3 −0.239X1X2−0.070X1X3−0.478X2X3 −0.012X1²+6.655X2²−0.101X3²,

(5) Yu1SR=−27.358+3.022X1+9.793X2+1.488X3 −6.251X1X2−0.072X1X3−0.487X2X3 −0.0740X12+9.506X22−0.022X32,

where *Yu*(SR), *Yu*_1_(SR), *X*_1_, *X*_2_, and *X*_3_ represent surface roughness of aqueous NaNO_3_, Cu nanoparticles suspended in aqueous NaNO_3_ electrolyte, applied voltage, tool feed rate, and electrolyte discharge rate, respectively. The coefficient of determination *R* ^2^ obtained from ANNOVA for surface roughness in terms of aqueous NaNO_3_ and Cu nanoparticles suspended in aqueous NaNO_3_ electrolytes were 92.55% and 96.98%, respectively, which confirms the fitness of the mathematical model.

## 4. Optimization Using Multiobjective Genetic Algorithm in MATLAB

Evolutionary algorithms seem mainly suitable to solve multiobjective optimization problems, because they deal simultaneously with a set of possible solutions (population). The traditional mathematical programming techniques need a series of separate runs to find the optimum solution for multiobjective problems. Contrarily, this method allows finding several members of the optimal set in a single run of the algorithm. In this research work multiobjective genetic algorithm toolbox from the MATLAB software is chosen for optimizing the selected objectives, maximizing the MRR and minimizing the surface roughness. The ability of GA to simultaneously search different regions of a solution space makes it possible to find a diverse set of solutions for difficult problems with nonconvex, discontinuous, and multimodal solutions spaces [13–15].

## 5. Analysis of the Influence of Parametric on the MRR and Surface Roughness for Aqueous NaNO_**3**_ Electrolyte

The mathematical models developed using RSM and presented in (2) and (4) were used in GA toolbox as fitness functions. The limitation for the optimization is given as follows:

(6) $$\begin{array}{c} 6\leq x_{1}\geq 12,\;\;3\leq x_{2}\geq 9,\;\;3\leq x_{3}\geq 9. \end{array}$$

The GA generally includes three fundamental genetic operations of selection, namely, population, crossover, and mutation. These operations are used to modify the chosen solutions and select the most appropriate offspring to pass on to succeeding generations. The following parameters were considered during optimization using GA multiobjective tool. Population size = 225, crossover fraction = 0.8, mutation function = constraint dependent, crossover function = scattered, and number of iterations = 188.

**Figure 2:**
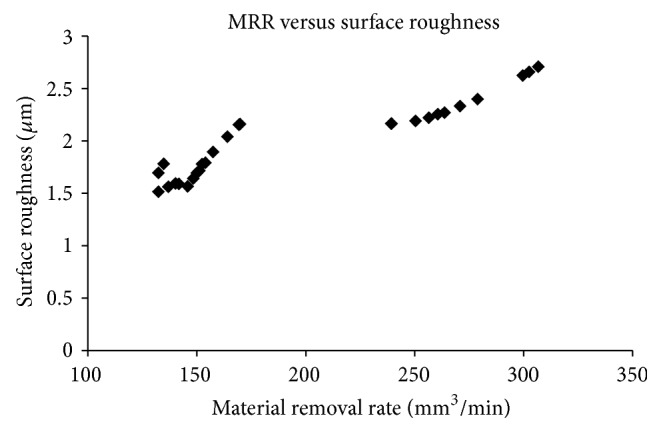
Optimal parameters of aqueous NaNO_3_ solution from GA.

Upon applying objective functions in GA tool, the results were obtained as tabulated in Table 3 and Figure 2.

The response plot shows the effects of applied voltage, tool feed rate, and electrolyte discharge rate on MRR and surface roughness of HCHCr die tool steel. MRR increases at higher voltage with the increase of tool feed rate and higher flow of electrolyte discharge rate at a mean time surface roughness slightly increased. A maximum MRR 306.69449 mm^3^/min was achieved under tool feed rate of 0.5399502 mm/min, 11.97976 lit/min of electrolyte discharge rate, and applied voltage of 17.995820 V. A minimum SR value of 1.513575 *µ*m was observed at 12 V, 0.1100281 mm/min of tool feed rate, and 8.134412 lit/min of electrolyte discharge rate.

## 6. Analysis of the Influence of Parametric on the MRR and Surface Roughness for Cu Nanoparticles Suspended in Aqueous NaNO_**3**_ Electrolyte

**Figure 3:**
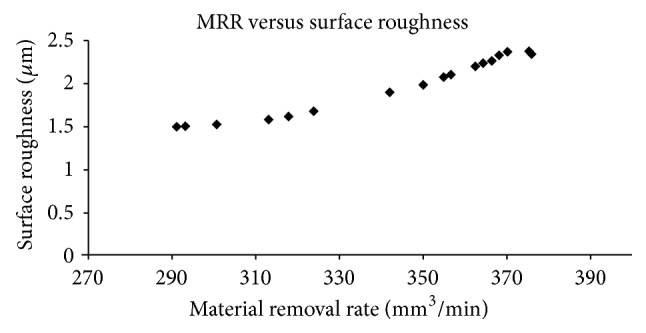
Optimal parameters of Cu nanoparticles suspended in aqueous NaNO_3_ solution from multiobjective GA.

Table 4 and Figure 3 present the results from GA for Cu nanoparticles suspended in aqueous NaNO_3_ electrolyte. MRR increases at higher values of electrolyte discharge rate and tool feed rate. The surface roughness decreases when the electrolyte discharge rate and tool feed rate are decreased. A maximum value of MRR 375.78277 mm^3^/min was obtained under 17.688986 V, 0.5399705 mm/min tool feed rate, and 11.998816 lit/min electrolyte discharge rate conditions. The minimum surface finish of 1.4973965 *µ*m was observed at 17.999473 V, 0.2344207 mm/min tool feed rate, and 11.997052 lit/min electrolyte discharge rate condition.

It is obvious that the optimum search can be obtained based on the developed second-order response, surface equations for correlating the various process variable effects with the MRR and surface roughness. The optimal combination of various process variables thus obtained for achieving controlled electrochemical machining of the workpieces is found to be within the bounds of the mathematical model.

## 7. Confirmation Test

The confirmatory experiments were further conducted for the optimal parameters obtained from the MATLAB multiobjective GA tool. The error between optimum values from GA and the confirmation test was derived by considering the serial number 24 and serial number 1 from Tables 3 and 4, respectively, at the condition of maximum MRR and is shown in Table 5.

## 8. Conclusions

This work employs a multiobjective genetic algorithm (GA) tool to optimize influencing parameters of ECM to maximize the MRR and minimize surface roughness of HCHCr die steel. Based on the experimental results, the following conclusions are drawn.

1. Material removal rate increases linearly with applied voltage and nonlinearly increases with tool feed rate. Surface roughness decreases with increase in the applied voltage and all tool feed rates. Mathematical models for MRR and surface roughness have been developed by Design Expert 7.0 software. It is useful for analyzing the influence of the various process parameters for achieving better MRR and surface roughness of HCHCr die tool steel.
2. Genetic algorithm (GA) tool optimizes the range of influencing parameters in order to obtain a maximum MRR and minimum surface roughness. The experimental results reveal that applied voltage of 18 V, tool feed rate of 0.54 mm/min, and electrolyte discharge rate of 12 lit/min would be the optimum values in ECM of HCHCr die tool steel under copper nanoparticles suspended in aqueous NaNO_3_ electrolyte solution machining condition.
3. For checking the optimality obtained from the multiobjective GA in MATLAB, MRR of 375.78277 mm^3^/min and surface roughness Ra of 2.339779 *μ*m were predicted at applied voltage of 18 V, tool feed rate of 0.54 mm/min, and electrolyte discharge rate of 11.99 lit/min.
4. Confirmatory tests showed that the actual performance at the optimum conditions was 361.214 mm^3^/min and 2.41 *μ*m; a deviation from the predicted performance is less than 4% at maximum material removal rate condition which has proven the composite desirability of the developed models for MRR and surface roughness under copper nanoparticles suspended in aqueous NaNO_3_ electrolyte solution machining condition. Aqueous NaNO_3_ electrolyte solutions performance is poor comparing to copper nanoparticles suspended in aqueous NaNO_3_ electrolyte solution.
5. Comparing the predicted performance of aqueous NaNO_3_ and copper nanoparticles suspended in aqueous NaNO_3_ electrolyte solutions on experimentally and mathematically, copper nanoparticles suspended in aqueous NaNO_3_ electrolyte solution performs better for MRR and surface roughness on HCHCr die tool steel.

## Figures and Tables

**Table 1:** Chemical composition of HCHCr die tool steel.

Element	C	Cr	Mn	P	S	Fe	Si
Wt%	1.936	11.84	0.27	0.044	0.089	85.34	0.48

**Table 2:** Process parameters.

Applied voltage (V)	12, 15, and 18
Interelectrode gap (mm)	0.1
Tool feed rate (mm/min)	0.1, 0.21, 0.32, and 0.54
Electrolyte discharge rate (lit/min)	8, 10, and 12
Selected electrolyte	15% NaNO_3_ aqueous solution 40 g copper nanoparticles suspended in 15% NaNO_3_ aqueous solution
Tool-electrode condition	Stationary
Electrolyte temperature range (°C)	30°–40°
Workpiece material with its hardness	HCHCr die steel—67 HRc
Machining time (min)	3

**Table 3:** Process decision variables along with optimized response from GA for aqueous NaNO_3_.

Sl. number	Voltage (V)	Feed rate (mm/min)	Discharge rate (lit/min)	MRR (mm^3^/min)	Surface roughness (micron)
1	12	0.1100281	8.134412	132.46309	1.513575
2	12.030211	0.1104329	9.087866	152.49089	1.777581
3	12.014045	0.1039534	10.04123	169.34532	2.152654
4	17.995146	0.5265484	11.98002	302.51291	2.656720
5	12.000245	0.1005786	9.147932	153.98288	1.791250
6	17.991590	0.3675932	11.97936	260.68191	2.254694
7	12.006746	0.2003393	8.234414	137.00311	1.560310
8	12.008757	0.1019808	10.06793	169.87964	2.159093
9	17.989152	0.3175749	11.99351	250.47074	2.189059
10	12.000737	0.1004096	9.719882	164.05383	2.038806
11	12.009474	0.2017167	8.488291	141.77756	1.587574
12	17.994398	0.5169848	11.97913	299.58104	2.623729
13	17.985153	0.2530979	11.99503	239.31519	2.164326
14	17.988464	0.3482414	11.99409	256.55458	2.219778
15	12.012065	0.1043002	8.710513	145.82497	1.564311
16	17.990082	0.4115904	11.98257	270.80421	2.330752
17	17.986068	0.3813624	11.99307	263.68107	2.268322
18	12.004311	0.1041213	8.848672	148.38304	1.639634
19	12.000057	0.2000266	8.109496	134.59464	1.771653
20	12.015943	0.1038107	8.938570	150.04776	1.691262
21	17.988003	0.4437773	11.99324	278.90557	2.397192
22	12.001201	0.1004778	8.995683	151.23304	1.714381
23	12	0.2	8.874121	132.46309	1.693575
24	17.995820	0.5399502	11.97976	306.69449	2.706127
25	12.007139	0.1076954	8.422892	140.26985	1.592297
26	17.985182	0.2532877	11.99497	239.34474	2.164350
27	12.013497	0.1039507	9.351800	157.47043	1.892639

**Table 4:** Process decision variables along with optimized response from GA for Cu nanoparticles suspended in aqueous NaNO_3_ electrolyte.

Sl. number	Voltage (V)	Feed rate (mm/min)	Discharge rate (lit/min)	MRR (mm^3^/min)	Surface roughness (micron)
1	17.688986	0.5399705	11.998816	375.78277	2.339779
2	17.999473	0.2344207	11.997052	291.21779	1.4973965
3	17.982536	0.3619794	11.990806	324.01735	1.6773238
4	17.812326	0.5399910	11.998295	375.72198	2.3501116
5	17.974140	0.4719105	11.991869	354.97140	2.0744706
6	17.986820	0.3385383	11.997917	317.93316	1.6169858
7	17.995289	0.2727332	11.99707	300.7935	1.5171501
8	17.981729	0.4545162	11.997314	350.07699	1.9885303
9	17.970973	0.5100869	11.997924	366.45561	2.2630915
10	17.991024	0.3212817	11.997223	313.33371	1.5817197
11	17.957900	0.5030484	11.997998	364.38746	2.2357070
12	17.955889	0.4263774	11.997138	342.11877	1.8942504
13	17.896183	0.5389892	11.998794	375.34015	2.3713213
14	17.917751	0.5150918	11.998277	368.07683	2.3296118
15	17.960839	0.4768214	11.996795	356.60081	2.1042235
16	17.913224	0.5219700	11.998223	370.14799	2.3709887
17	17.963386	0.4965703	11.997834	362.44458	2.1984027
18	17.997099	0.2431022	11.997066	293.37153	1.5003086
19	17.991022	0.3212817	11.997223	313.33371	1.5817197

**Table 5:** Error between optimum values from GA and confirmation test value for maximum MRR.

Sl. number	Electrolyte	Obtained from GA		Confirmation test		Error	
		MRR mm^3^/min	SR *μ*m	MRR mm^3^/min	SR *μ*m	MRR %	SR %
1	Aqueous NaNO_3_	306.69449	2.706127	294.012	2.82	4.13	4.25
2	Cu nanoparticles suspended in aqueous NaNO_3_	375.78277	2.339779	361.214	2.41	3.87	3.31

## References

[1] Ahn S. H., Ryu S. H., Choi D. K., Chu C. N. (2004). Electro-chemical micro drilling using ultra short pulses. *Precision Engineering*.

[2] Goswami R., Chaturvedi V., Chouhan R. (2013). Optimization of electrochemical machining process parameters using Taguchi approach. *International Journal of Engineering Science and Technology*.

[3] Sekar T., Marappan R. (2008). Experimental investigations into the influencing parameters of electrochemical machining of AISI 202. *Journal of Advanced Manufacturing Systems*.

[4] Tang L., Guo Y.-F. (2013). Experimental study of special purpose stainless steel on electrochemical machining of electrolyte composition. *Materials and Manufacturing Processes*.

[5] Li Z., Ji H. Machining accuracy prediction of aero-engine blade in electrochemical machining based on BP neural network.

[6] Wang M., Peng W., Yao C., Zhang Q. (2010). Electrochemical machining of the spiral internal turbulator. *International Journal of Advanced Manufacturing Technology*.

[7] Hasçalik A., Çaydaş U. (2007). A comparative study of surface integrity of Ti-6Al-4V alloy machined by EDM and AECG. *Journal of Materials Processing Technology*.

[8] Brusilovski Z. (2008). Adjustment and readjustment of electrochemical machines and control of the process parameters in machining shaped surfaces. *Journal of Materials Processing Technology*.

[9] Kozak J., Chuchro M., Ruszaj A., Karbowski K. (2000). The Computer aided simulation of electrochemical process with universal spherical electrodes when machining sculptured surfaces. *Journal of Materials Processing Technology*.

[10] Judal K. G., Yadava V. (2013). Cylindrical electrochemical magnetic abrasive machining of AISI-304 stainless steel. *Materials and Manufacturing Processes*.

[11] Tan P. C., Yeo S. H. (2011). Investigation of recast layers generated by a powder-mixed dielectric micro electrical discharge machining processg. *Proceedings of the Institution of Mechanical Engineers, Part B: Journal of Engineering Manufacture*.

[12] Bhattacharyya B., Malapati M., Munda J. (2005). Experimental study on electrochemical micromachining. *Journal of Materials Processing Technology*.

[13] Jones D. F., Mirrazavi S. K., Tamiz M. (2002). Multi-objective meta-heuristics: an overview of the current state-of-the-art. *European Journal of Operational Research*.

[14] Tang K., Yang J., Chen H., Gao S. (2011). Improved genetic algorithm for nonlinear programming problems. *Journal of Systems Engineering and Electronics*.

[15] Mukherjee R., Chakraborty S. (2013). Selection of the optimal electrochemical machining process parameters using biogeography-based optimization algorithm. *The International Journal of Advanced Manufacturing Technology*.

